# Model of Liver Fibrosis Induction by Thioacetamide in Rats for Regenerative Therapy Studies

**DOI:** 10.1155/2022/2841894

**Published:** 2022-11-12

**Authors:** Nathaly Enciso, José Amiel, Fredy Fabián-Domínguez, Jhon Pando, Nancy Rojas, Carlos Cisneros-Huamaní, Ernesto Nava, Javier Enciso

**Affiliations:** ^1^Grupo de Medicina Regenerativa, Universidad Científica del Sur, Lima 150142, Peru; ^2^Dirección General de Investigación, Desarrollo e Innovación, Universidad Científica del Sur, Lima 150142, Peru; ^3^Investigador Adjunto, Grupo de Medicina Regenerativa, Universidad Científica del Sur, Lima 150142, Peru; ^4^Instituto de Criopreservación y Terapia Celular, Lima 15074, Peru; ^5^Laboratorio de Microscopía Electrónica, Universidad Nacional Mayor de San Marcos, Lima 506, Peru

## Abstract

Hepatic fibrosis is caused by chronic injury due to toxic, infectious, or metabolic causes, and it may progress to cirrhosis and hepatocellular carcinoma. There is currently no antifibrotic therapy authorized for human use; however, there are promising studies using cell therapies. There are also no animal models that exactly reproduce human liver fibrosis that can be used to better understand the mechanisms of its regression and identify new targets for treatment and therapeutic approaches. On the other hand, mesenchymal stem cells (MSC) have experimentally demonstrated fibrosis regression effects, but it is necessary to have an animal model of advanced liver fibrosis to evaluate the effect of these cells. The aim of this work was to establish a protocol for the induction of advanced liver fibrosis in rats using thioacetamide (TAA), which will allow us to perform trials using MSC as a possible therapy for fibrosis regression. For this purpose, we selected 24 female rats and grouped them into three experimental groups: the control group (G-I) without treatment and groups II (G-II) and III (G-III) that received TAA by intraperitoneal injection for 24 weeks. Then, 1 × 106/kg adipose mesenchymal stem cells (ASCs) were infused intravenously. Groups G-I and G-II were sacrificed 7 days after the last dose of ASC, and G-III was sacrificed 8 weeks after the last ASC infusion, all with xylazine/ketamine (40 mg/kg). The protocol used in this work established a model of advanced hepatic fibrosis as corroborated by METAVIR tests of the histological lesions; by the high levels of the markers *α*-SMA, CD68, and collagen type I; by functional alterations due to elevated markers of the hepatic lesions; and by alterations of the leukocytes, lymphocytes, and platelets. Finally, transplanted cells in the fibrous liver were detected. We conclude that TAA applied using the protocol introduced in this study induces a good model of advanced liver fibrosis in rats.

## 1. Introduction

Chronic liver disease (CLD) is a continuous process of inflammation, destruction, and regeneration of the liver parenchyma, leading to fibrosis and cirrhosis resulting in progressive deterioration of liver function. The spectrum of etiologies is broad for chronic liver disease, including toxins, long-term alcohol abuse, infections, autoimmune diseases such as primary biliary cirrhosis, primary sclerosing cholangitis, and autoimmune hepatitis, and genetic and metabolic disorders [1, 2]. CLD and cirrhosis are responsible for 2 million deaths per year worldwide [3], while an estimated 1.5 billion people worldwide suffer from chronic liver fibrosis which is a health burden [4].

Chronic liver disease is characterized by fibrosis, which is the accumulation of extracellular matrix (ECM) proteins and chronic inflammation. ECM is identified by immune cells as damage-associated molecular patterns (DAMPs) with immunologically active peptides and domains. During liver injury, ECM deposition and remodeling as well as fibrosis can lead to the formation of DAMPs, which would trigger a local inflammatory immune response and recruitment of immune cells [5].

Liver fibrosis is potentially reversible, and it can be stopped from progressing to cirrhosis [6]. Many candidate antifibrotic agents have shown promising effects in experimental animal models; however, their antifibrotic effects in clinical trials have been limited, so there is currently no approved specific therapy for chronic liver fibrosis [7].

Despite current advances, the treatment of chronic liver failure remains one of the most critical problems in clinical medicine. Therefore, the availability of suitable experimental models is of crucial importance to better understand this disease, to allow the identification of new therapeutic targets and thus to test efficacy and evaluate mechanisms of toxicity [8].

The main therapy for end-stage liver fibrosis is liver transplantation. However, the shortage of healthy livers, complex surgical procedures, and high costs are driving researchers to develop new approaches to treat liver fibrosis before it progresses. Mesenchymal stem cell (MSC) therapy is a promising alternative approach for the treatment of patients with chronic liver fibrosis. However, many aspects of this therapy, such as efficacy compared to conventional treatment, remain unclear. [9, 10], so it is necessary to establish a model of advanced chronic fibrosis that allows for stem cell xenotransplantation and monitoring of its effects and mechanisms of action.

Experimental hepatotoxicity in rodents with thioacetamide has been studied since 1948 [11]. Despite this, very few rodent models develop advanced fibrosis and, at the same time, reflect the etiology or pathology of the disease in humans, which is problematic. Fibrosis stage is considered the best prognostic marker in patients and is an important endpoint in fibrosis clinical trials [12]. Currently, no animal model is available that can recapitulate all of the hepatic and extrahepatic features of liver disease [13], although various doses and protocols have been used to develop different animal models [14–21].

In this work, we developed a model of chemical hepatotoxicity by TAA associated with advanced fibrosis and chronic liver failure. We then applied a new protocol of induction and xenotransplantation of stem cells, without producing high mortality, and used our rat model to perform assays of the response and mechanisms of action of human mesenchymal stem cell xenotransplantation.

## 2. Materials and Methods

### 2.1. Animals

Twenty-four female Holtzman rats, 7 weeks old and weighing 180-200 g, were acquired at the National Institute of Health of Peru (INS) and were maintained for 2 weeks in the biotherium of the Scientific University of the South (UCSUR) under stable environmental conditions. Furthermore, the rats used in this study were treated according to the norms established by the INS, the European Community and Spanish legislation (Real Decreto 53/2013) for care and welfare of laboratory animals. All protocols were approved by the Ethics Committee of the Veterinary Faculty, UCSUR (approval number 005-2013).

### 2.2. Experimental Groups and the Induction of Chronic Fibrosis

This experimental design was based on previous work [10, 22], which in summary is described below. To induce advanced chronic fibrosis, 3 experimental groups were formed: G-I, G-II, and G-III, with 8 animals each. G-I was the control group that received intraperitoneal (IP) treatment with 24 doses of saline once a week, while G-II and G-III received 24 doses of 200 mg/kg thioacetamide diluted in 2.2 mL of 0.9% sodium chloride that were delivered intraperitoneally once a week. G-II animals were euthanized seven days after the application of the last dose of TAA by pretreatment with xylazine/ketamine (40 mg/kg) and subsequent saturation with chloroform. Once it was confirmed that GII showed features of chronic liver fibrosis, G-III received an intravenous dose of human ASCs 7 days after the last dose of TAA, and at the end of 8 weeks, the rats were sacrificed to investigate their liver and kidney structure and function and to detect the presence of the transplanted cells.

### 2.3. Histopathology

After euthanasia, necropsy was performed for morphological study of the liver, the description of which was recorded in a format designed for this purpose. A fresh 0.5 × 1 cm liver sample was taken and fixed in 10% formaldehyde solution (formalin) in PBS (pH 7.4), changing the fixative solution to fresh solution after 24 hours, then it was sent for histological processing by embedding in paraffin, cutting into 5 *μ*m thick sections with a microtome and finally stained with hematoxylin and eosin (H&E) and Masson's trichrome (M-T). Samples were observed under a NIKON Eclipse E200 microscope; NIS-ELEMENTS D3.1 imaging software was used for image analysis, and microphotographs were captured with a digital camera (CANON).

The grading of the histological lesions was based on the METAVIR test [23], which stages the degree of fibrosis and considers the following lesions indicating liver damage in general: proliferation of bile ducts, steatosis, dilatation of portal veins, fibrosis, and pigmented macrophages, which were graded as follows: F0 = no lesions (-), F1 = mild lesions (+), F2 = moderate lesions (++), and F3 = severe lesions (+++).

### 2.4. Human Adipose Stem Cells

To demonstrate that adipose stem cells (ASCs) could be transplanted into rats with chronic fibrosis without producing mortality, human ASCs of the 2nd passage were thawed at 37°C and centrifuged at 800 g to remove the supernatant and then expanded in DMEM-F12 with 10% fetal bovine serum (FBS) until the number required for the experiment was reached. From this population, an aliquot was taken for characterization by flow cytometry using the following panel of antibodies: CD19, CD90, CD34, CD105, and CD45. The experimental dose was 1 × 106 cells/kg body weight, [24] diluted in 300 *μ*l of phosphate buffer solution (PBS) and injected via the lateral tail vein with a 30 g × 1/2 inch needle.

### 2.5. Immunohistochemistry

To evaluate markers of chronic liver fibrosis and the presence of adipose stem cells in the liver, immunohistochemistry (IHQ) was used based on the protocols and techniques already described [25]. In brief, tissue samples were fixed in 10% buffered formalin and embedded in paraffin; after deparaffinization, they were subjected to an antigenic retrieval process by immersing them in citrate buffer (pH 6.8) and microwaving at a power of 6 for 5 minutes. Peroxidase blocking was performed with 30% hydrogen peroxide/methanol (1 : 9) for 10 minutes, and then the slides were incubated with the primary antibody in a humidity chamber for 1 hour at room temperature or overnight at 4°C. The primary antibodies were against *α*-SMA, CD68, and collagen type I (INVITROGEN) to identify chronic liver fibrosis and HuNu (human anti-nucleotide antibody, clone 235-1. Sigma, Aldrich) to identify human ASCs. A biotinylated secondary antibody was then added for 20 min followed by HRP for 10 minutes, and diaminobenzidine (DAB) (INVITROGEN) was applied as a chromogen for 1-3 minutes.

### 2.6. Biochemistry/Blood Count

Whole blood was obtained immediately after euthanasia for hemogram and biochemical analysis. The hemogram was performed with an RT-7600 autohematology analyzer (Rayto), while an RT-1904CV automated analyzer (Rayto) was used for biochemical analysis. The analytes were as follows: *α*-fetoprotein, prothrombin, glucose, cholesterol, aspartate aminotransferase (AST), alkaline phosphatase (ALP), alanine aminotransferase (ALT), urea, blood urea nitrogen (BUN), total protein, albumin, globulin, direct bilirubin (BD), total bilirubin (BT), and indirect bilirubin (BI).

### 2.7. Statistics

Data were analyzed using Graph Pad Prism version 6 and SPSS 25 software (IBM Corporation, Endicott, NY, USA). Shapiro-Wilk test was used to evaluate the normal distribution of the data. ANOVA test with post-Tukey's multiple comparisons test was used for evaluating Biochemical and hemogram analysis data between rats with advanced chronic fibrosis undergoing to no treatment or ASC treatment after euthanasia. All data are expressed as the mean ± SD. It was considered statistically significant when *p* < 0.05.

## 3. Results

### 3.1. Adipose Mesenchymal Stem Cells

**Figure 1:**
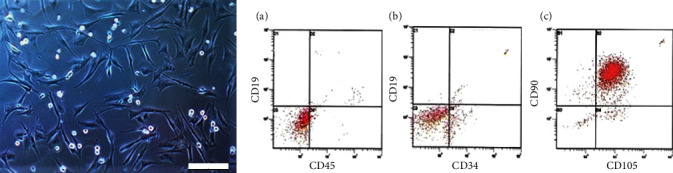
(a) Representative photographs of adipose mesenchymal adipose stem cells from 2^nd^ passage showed spindle-shaped fibroblastic cell morphology. Bar length = 200 *μ*m. (b) Representative dot plots of ASCs stained with the cell surface markers. ASCs have negative results for CD19, CD34, and CD45 ((b) A, B) and positive results for CD90 and CD105 ((b), C).

The ASCs showed a fibroblastic cellular morphology attached to the plate. (Figure 1(a)). Using a panel of markers representative of human ASCs, we found that they expressed the previously described markers by flow cytometry. The cells were positive for CD90 and CD105 and negative for CD19, CD34, and CD45 (Figure 1(b)).

### 3.2. Body Weight of the Rats

**Figure 2:**
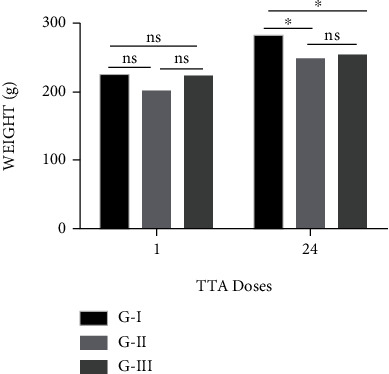
Average weight (g) of rats during the chronic fibrosis induction period after treatment with 24 doses of TAA, n : 8. (a) Bar graph of the weight of the rats at the beginning and end of treatment. (b) ^∗^*p* < 0.05; ns: not significant.

After 24 doses of thioacetamide, the G-II and G-III rats presented weight losses of 11.83% and 10.22%, respectively, compared to the control group (Figure 2), and there was a significant difference after 24 weeks compared to G-I. At the end of the experiment, there was a mortality of 15%.

### 3.3. Macroscopic Morphopathology

**Figure 3:**
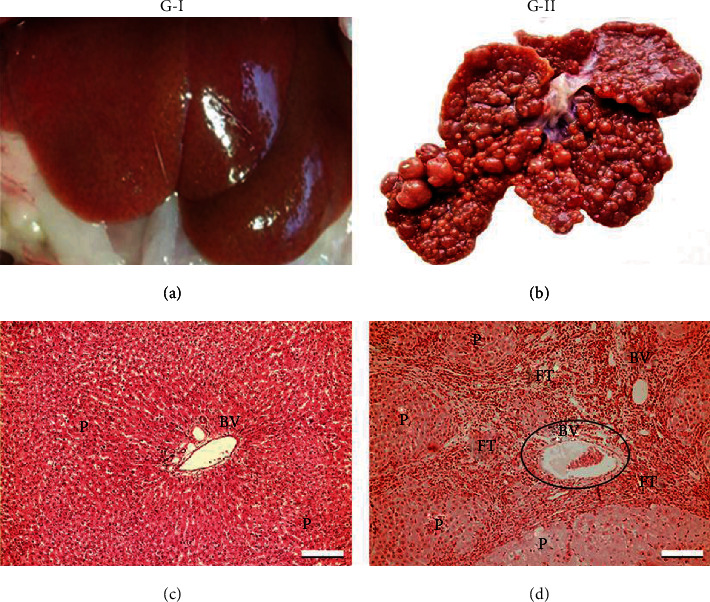
Representative photographs of liver histology. G-I control, (a) liver shows reddish brown colour, (c) without histological lesions. G-II treatment with TAA, (b) liver shows multinodular tissue, (d) increased fibrous tissue (blue arrows) and few areas of liver parenchyma. P: parenchyma; VS: blood vessel; TF: fibrous tissue, H&E. Bar length = 200 *μ*m.

At the end of the trial, 100% (n : 8) of the animals in group G-II showed fibrotic liver, with irregular borders and multiple macro and micronodules, and no lesions were observed in the spleen (Figure 3). In G-III, after treatment with adipose mesenchymal stem cells, a moderate decrease in the number of nodules was observed in 62.5% (5/8) of rats and a slight increase in liver, and spleen weight were observed compared to G-II (Table 1).

### 3.4. Histopathology

**Figure 4:**
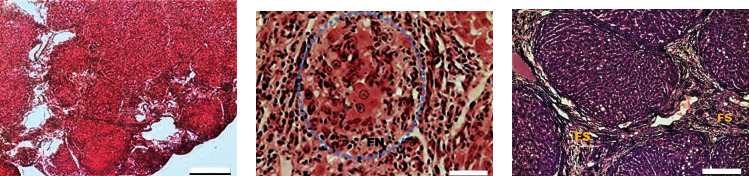
Representative photographs of liver of group G-II. (a) Chronic fibrosis, H&E staining. (b) Fibrotic nodule (FN), H&E staining. (c) Fibrosis septa (FS) by Masson's staining. Bar length = 400 *μ*m (a), 50 *μ*m (b), and 200 *μ*m (c).

The histological study showed an increase in fibrous tissue with blood vessels and a few areas of liver parenchyma in G-II compared to the control (G-I), which did not receive TAA (Figure 2). In most animals (7/8 rats), advanced fibrosis (++ and +++), remarkable proliferation of bile ducts (++), mild steatosis, and pigmented macrophages predominated (Table 2). The fibrotic nodules (FN) (Figure 4(b)) were surrounded by fibrous septa (FS), as shown by Masson's staining (Figure 4(c)). Some septa were connected between the portal tract and the central vein. Fibrous septa were observed in the areas where the bile ducts proliferated. In the portal space, there were increased fibrous tissue, periportal veins, and Kupffer cell hyperplasia in the parenchyma, and all of these lesions corresponded to advanced fibrosis (Figure 4). When measured by the METAVIR score, group II animals that received TAA by IP injection had lesions compatible with grade F3 (n : 2), F2 (n : 5), and F1 (n : 1) (Table 3).

### 3.5. Liver Fibrosis Markers

**Figure 5:**
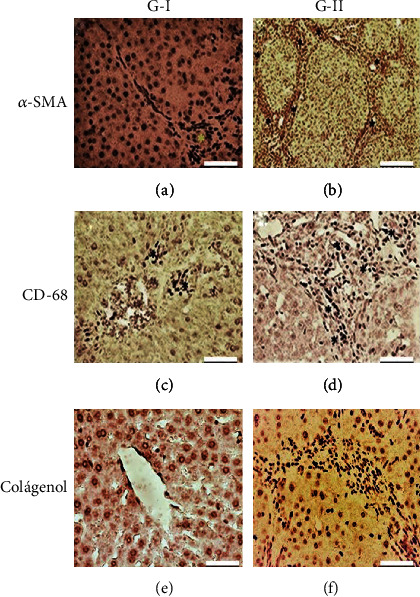
Molecular markers of chronic liver fibrosis: *α*-SMA, CD68, and Collagen I. (a, c, and d) G-I control liver and (b, d, and f) G-II liver with chronic liver fibrosis, IHC. Bar length = 50 *μ*m. ^∗^: identifies the immunoreactivity of the cells to the specific marker.

IHC confirmed the presence of chronic liver fibrosis in G-II, showing a higher population of *α*-SMA+ cells, CD68+, and type I+ collagen, which are markers of chronic liver fibrosis, than in the control group (Figure 5).

### 3.6. Immunodetection of Adipose Stem Cells

**Figure 6:**
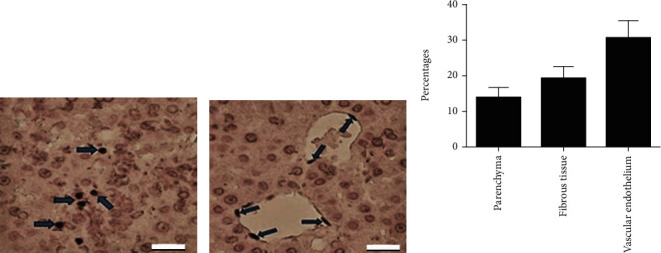
Adipose stem cells in different areas of the liver (blue arrows). (a) liver parenchyma. (b) endothelium, IHC. (c) Percentages of positive immunoreactivity to HuNu antibody. Bar length =50 *μ*m.

Eight weeks after inoculation of human adipose stem cells in G-III rats, these cells could be detected in the liver parenchyma, fibrous tissue, and vascular endothelium at different percentages (Figure 6). When quantifying the presence of the transplanted human adipose stem cells in three topographical areas of the liver with chronic G-III fibrosis, there was a positive immunoreactivity to HuNu antibody of 13.77 ± 2.89 (SD) in parenchyma, 19.17 ± 3.26 (*SD*) in fibrous tissue, and 30.5 ± 4.84 (SD) in vascular endothelium.

### 3.7. Biochemistry and Hemogram Analysis

**Figure 7:**
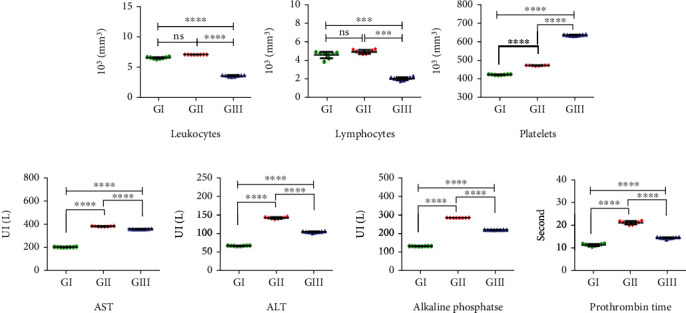
Effect of TAA on blood cells and on liver function. Comparative studies between blood count (a) and analytes values (b) of rats with chronic liver fibrosis, no treatment groups (GI and GII), and chronic liver fibrosis treated with ASCs (GIII). Data values are the mean ± SD. Representation of significant difference: ^∗∗∗∗^ is considered *p* < 0.001; ^∗∗∗∗^ is *p* < 0.0001, and ns = no significant difference.

We reported only the hematological parameters that showed statistically significant differences between the control (GI) and GII groups. As shown in Figure 7(a), total leukocytes and lymphocytes showed no significant difference (*p* value ≥0.05), while platelets increased significantly (*p* value <0.0001) in the group receiving TAA alone versus the control group. As shown in Figure 7(b), the analytes aspartate aminotransferase (AST) and alanine aminotransferase (ALT), alkaline phosphatase and prothrombin time increased significantly (*p* value <0.0001) in the group receiving TTA alone versus the control group.

On the other hand, although it was not the objective of this study, when comparing the group that received TAA plus ASC (G-III) with the control group (G-I), it was observed that there was a significant depletion of lymphocytes (*p* value <0.001) and total leukocytes (*p* value <0.0001) as well as a very significant increase (*p* value <0.0001) in platelets in G-III. However, when comparing G-II with G-III, a highly significant decrease in AST, ALT, ALP, and prothrombin (*p* value <0.0001) was observed in G-III (data not shown in this work).

## 4. Discussion

The present work demonstrated that 24 once-weekly doses of 200 mg/kg IP injection of TAA induced advanced chronic fibrosis with similar histological lesions in all rats in this trial. They were histological and functional lesions with abundant cell populations with positive reactivity to the fibrosis markers *α*-SMA, CD68, and type I collagen. Other authors reported similar results, such as patterns of parenchymal fibrosis [26], pericellular/perisinusoidal fibrosis, and centrilobular fibrosis [27]. In this work, we applied the METAVIR test, which measures the degree of inflammatory activity [23] and found that all animals that received TAA developed fibrosis.

Wallace et al. [22] reported a mortality higher than 15% using TAA via IP in rats at a dose of 150 mg/kg diluted in 1-1. 5 mL of 0.9% sodium chloride (NaCl), three times per week for a total of 11 weeks, while we had a lower mortality (15%) despite using a higher dose of TAA, 200 mg/kg diluted in 2.2 mL of 0.9% NaCl, but once per week for 24 weeks. This would indicate that with our protocol we would obtain chronic liver fibrosis but with lower mortality despite using a higher dose.

It is the only article that presents TAA treatment for a longer period of time than the others; furthermore, the chronic hepatic fibrosis induced in this work was histologically characterized by the formation of extensive areas of fibrous tissue distributed between the alveolar septa and central veins as shown by H&E and Masson's staining. These histological features are important for the fibrosis model we are looking for, as it has been shown that TAA-induced fibrosis persists for more than 2 months after the last dose [10], unlike that produced by carbon tetrachloride (CCL4) [28], and it is associated with more prominent regenerative nodules and rapid development of portal fibrosis, a state that resembles human cirrhosis [15]. Unlike the model using carbon tetrachloride, in this model with TAA, no bacterial overgrowth has been described that may influence the development of fibrosis/cirrhosis [29], thus representing a model of advanced chronic fibrosis that does not spontaneously reverse early, allowing for tests of cell therapy that might have reversal effects on this type of liver injury.

Functionally, chronic liver fibrosis was induced by TAA according to our protocol, as demonstrated by a statistically significant increase in the biochemical markers AST, ALT, alkaline phosphatase, and prothrombin. This demonstrates the efficacy of the protocol, and it has lower mortality than that reported by other authors using CCL4 [30, 31]. Likewise, there are no differences in total leukocytes and lymphocytes, which is helpful for conducting stem cell assays since these cells are immunosuppressive, producing severe lymphopenia in the treated animals [32], as was the case in group GIII, which showed a decrease in the total number of lymphocytes compared to GII, demonstrating the effect of mesenchymal cells in animals treated with these cells.

On the other hand, in our model, a significant increase in platelets was observed, which does not occur with other fibrosis inducers like CCL4, which instead develop thrombocytopenia [31]. We do not have an explanation to support this difference, but we hypothesize that perhaps, during the processing and performance of the test, there were some alterations of the sample. Future works can elucidate this issue.

The protocol of this work demonstrated that this model of advanced liver fibrosis induced by TAA allows for evaluation of possible antifibrotic effects of cell therapy. We showed that human adipose stem cells infused in animals with advanced liver fibrosis infiltrated the affected organ without producing adverse effects. By means of IHC, we demonstrated the presence of human adipose stem cells in the liver parenchyma and stroma at 8 weeks post infusion without causing any mortality. This knowledge will allow us to perform future trials to evaluate the optimal number of cells per dose, dose repetitions, and types of transplanted cells and to determine their mechanisms of action.

## 5. Conclusions

Advanced hepatic fibrosis in rats can be induced by 24 doses once a week of 200 mg/kg IP injection of TAA. The rats develop macroscopic lesions represented by multiple macro and micronodules, while the histological study showed abundant fibrous septa. Immunohistochemistry detected abundant *α*-SMA, CD-68, and type I collagen, which are markers of hepatic fibrosis, while functionally, elevated levels of AST, ALT, alkaline phosphatase, and prothrombin were present. Finally, human adipose stem cells were detected by IHC in the parenchyma, fibrous tissue, and vascular endothelium 8 weeks after infusion. However, to explain the mechanisms by which tissue is regenerated as well as to determine whether booster doses are necessary, further research is still needed.

## Figures and Tables

**Table 1:** Presence of nodules and weight of liver and spleen at necropsy according to experimental groups.

Groups	Liver						Spleen	
	Nodules					Weight	Lesions	Weight
	Absence	Regular	Abundant	Ma^1^	Mi^2^	(g)		(g)
I	8 rats	—	—	—	—	7	N^3^	0.67
II	—	—	8 rats	Yes	Yes	9.87	N	0.94
III	—	5 rats	3 rats	Yes	Yes	10.12	N	1.24

(1) Ma: macronodules, (2) Mi: micronodules, and (3) N: none.

**Table 2:** Grading of histological lesions present in group G-II liver fibrosis.

N	Fibrosis		Histological lesions				
	Yes	No	Bile duct proliferation	Steatosis	Dilatation portal veins	Fibrosis	Macrophage with pigments
1	+	—	++	+	+	+++	+
2	+	—	++	—	+	+	+
3	+	—	++	+	+	++	+
4	+	—	++	+	+	++	+
5	+	—	++	+	+	++	+
6	+	—	++	+	+	++	+
7	+	—	++	++	+	++	+
8	+	—	++	+	+	+++	+

**Table 3:** Grading of histological lesions according to experimental groups using the METAVIR score.

Phase	Anatomopathological characteristic	Number of animals per experimental groups	
		Group I	Group II
F0	No fibrosis	8	0
F1	Portal fibrosis without septa	0	1
F2	Portal fibrosis with few septa	0	5
F3	Portal fibrosis with numerous septa	0	2
F4	Cirrhosis	0	0

## Data Availability

All data used to support the findings of this study are included within the article.

## Additional Points

*Impact statement*. There is currently no antifibrotic therapy authorized for human use. The treatment of advanced liver fibrosis with stem cells is accepted as a good therapeutic alternative; however, the heterogeneity of the preclinical models used makes it difficult to compare the results objectively across studies. This work proposes a new protocol for the induction of advanced liver fibrosis in rats by 24 doses of 200 mg/kg thioacetamide, which in our work produces low mortality and uses conventional tests and markers (METAVIR test, *α*-SMA, CD68, collagen type I, ALT, alkaline phosphatase, and others) to establish the stage of the disease and the regression of the fibrosis. Stem cells can be detected in the hepatic parenchyma, 8 weeks postinfusion. We believe that this simple protocol would allow us to functionally and molecularly evaluate the effect of human stem cell therapy and its secretome on advanced fibrosis in a rat model.
